# LPS Administration during Fertilization Affects Epigenetic Inheritance during Embryonic Development

**DOI:** 10.3390/ani13071135

**Published:** 2023-03-23

**Authors:** Sangwoo Kim, Erina Yoneda, Kisaki Tomita, Mitsunori Kayano, Hiroyuki Watanabe, Motoki Sasaki, Takashi Shimizu, Yuki Muranishi

**Affiliations:** Graduate School of Animal and Veterinary Sciences and Agriculture, Obihiro University of Agriculture and Veterinary Medicine, Hokkaido 080-8555, Japan

**Keywords:** embryo, endotoxin, infertility, inflammatory, LPS

## Abstract

**Simple Summary:**

The long-term effect of exposure to lipopolysaccharide (LPS) endotoxins during fertilization in mammals has not been clarified. In this study, we examined the influence of LPS on early embryonic development and fetal development in mice. The uteruses of mice were examined for the expression of genes related to the inflammatory response. The expression of *Il-1β* and *Il-6* increased following the administration of 200 and 1000 µg/kg LPS. Exposure to LPS during in vitro fertilization (IVF) significantly decreased the embryonic developmental rate. A concentration of 100 µg/kg LPS significantly increased the placental weight and fetal crown–rump length (CRL), whereas a concentration of 200 µg/kg LPS significantly decreased the placenta weight and fetal weight in vivo at 18.5 days post-coitus (dpc). In summary, this study demonstrated that LPS exposure during fertilization causes abnormal embryonic phenotypes and fetal development in mice. Maternal endotoxins may affect epigenetic inheritance in embryonic development from the early to late stages of pregnancy.

**Abstract:**

Intrauterine inflammation can cause infertility by disrupting reproductive function. The pathogenesis underlying this process may primarily involve endotoxins from lipopolysaccharides (LPS), which are produced by Gram-negative bacteria. However, the long-term effects of endotoxins in mammalian pregnancy following LPS exposure during fertilization have not been clarified. In this study, we performed experiments to analyze the influence of LPS on early embryonic development and fetal development in mice. Mice uteruses were examined for the expression of genes related to the inflammatory response. The expression of *Il-1β* and *Il-6* increased following the administration of 200 and 1000 µg/kg LPS. Exposure to LPS using in vitro fertilization (IVF) significantly decreased the embryonic developmental rate. A concentration of 100 µg/kg LPS significantly increased the placental weight and fetal crown –rump length (CRL), whereas a concentration of 200 µg/kg LPS significantly decreased the placenta weight and fetal weight in vivo. These findings indicate that maternal LPS during fertilization affects fetal development until the late stage of pregnancy. Thus, maternal endotoxins may affect epigenetic inheritance during embryonic development from the early to late stages of pregnancy.

## 1. Introduction

Pregnancy is a complex process in which the maternal immune system has to tolerate the allogenic fetus to achieve a successful natal outcome [1]. Recently, several studies have focused on the relationship between disruption of the immune system balance in pregnancy by endotoxins and infertility and chronic intrauterine infections [2,3]. Endotoxins are a silent consequence of bacterial infections and are associated with various negative impacts on reproductive function in humans and livestock animals [4,5,6]. Generally, bacterial infections of the genital tract are linked to inflammatory disease in the uterus and ovaries [7]. Among bacteria that induce pathogenic infections, Gram-negative bacteria, such as *Escherichia coli*, secrete lipopolysaccharide (LPS) endotoxins. LPS consists of a lipid and carbohydrate chain and is one of the elements constituting the cell wall of Gram-negative bacteria. The carbohydrate chain consists of a hydrophilic core saccharide and an O-antigenic structure [8,9]. LPS is recognized by Toll-like receptor 4 (TLR4) on the cell surface, which forms a co-receptor with CD14 and MD2. The binding of LPS to TLR4 activates the nuclear signaling pathway for the transcription factor complex nuclear factor-kappa B (NF-*κ*B), which in turn produces pro-inflammatory cytokines and chemokines [8]. The production of LPS by bacteria during infection has been reported to trigger the expression of several genes, such as *IL-1β*, *IL-6,* and *TNF-α,* which are involved in inflammatory responses induced by TLR signaling pathways [10,11,12].

Studies have reported that LPS has harmful effects on the female genital tract [13] and intrauterine germ cells, namely the sperms and oocytes. In dairy cows, LPS decreased the mRNA expression of *StAR* and *CYP17*, gonadotropin receptors associated with steroid hormone cascades, and levels (concentration) of the steroid androgen and progesterone [14,15,16]. LPS levels were also correlated with the concentrations of PGE_2_ and E_2_ or P_4_ in ovarian follicles and uteruses with inflammatory uterine diseases [17,18,19,20]. In mice, LPS increased the mRNA expression of inflammatory cytokines *Il-6*, *Ptgs2*, and *Tnf-α* in cumulus cells [21]. Furthermore, late-stage LPS administration at 15 days post-coitus (dpc) increased the rate of abnormal fetal development [22]. In pigs, LPS decreased male reproductive performance by affecting sperm motility and sperm viability [23]. These studies suggest that endotoxemia might cause harmful immune responses during embryonic development in mammals.

However, whether maternal LPS exposure during fertilization directly affects the oocytes or acts via the uterus during pregnancy has not been clarified in mammals. Thus, the purpose of this study was to investigate the effect of LPS exposure at fertilization on early embryo and fetal development. We analyzed the effect of LPS on early embryonic development using a culture system of mice embryos and used fertilized mice as a mammalian model to evaluate the impact of LPS on pregnancy and fetal development.

## 2. Materials and Methods

### 2.1. LPS Treatment

LPS (*Escherichia coli* LPS, serotype O111:B4) was purchased from Sigma-Aldrich (St. Louis, MO, USA).

### 2.2. Animal Ethics and Care

The experimental procedures complied with the Guide for the Care and Use of Laboratory Animals by the Obihiro University of Agriculture and Veterinary Medicine (approval number 18-121, 22-173). The animals had free access to food and water throughout the experiment and were housed in a control room with a 12 h light/12 h dark cycle at a controlled temperature (23 ± 2 °C) and humidity (50 ± 5%).

### 2.3. Maternal Effect of LPS Administration in the Uterus

Female ICR mice (10–12 weeks old) were used to examine the effects of LPS administration on the uterus. Mice received an intraperitoneal (i.p.) injection of LPS at concentrations of 0, 10, 100, 200, or 1000 μg/kg. Then, 6 h after LPS administration the mice were sacrificed and blood samples were collected from the heart to measure the LPS concentration. Thereafter, the uterus was removed and washed in phosphate-buffered saline (PBS). Total RNA extracted from the uterus was used for gene expression analysis by immunoreaction. To analyze the immune response, the uterine color after LPS injection was measured using a CM-700d spectrophotometer (Konica Minolta, Tokyo, Japan). The color indicators included the following: L*: lightness, a*: redness, and b*: yellowness. Furthermore, to assess the morphology of the uterus, samples were fixed in 10% formalin (066-06821, Fujifilm Wako, Osaka, Japan), dehydrated using an ethyl alcohol series, embedded in paraffin, and stained with hematoxylin and eosin (H&E) staining. Images were obtained using a ZEISS Axio Zoom microscope.V16 for Biology (Carl Zeiss AG, Oberkochen, Germany) and the software ZEN 3.1 pro (Carl Zeiss AG).

### 2.4. Measuring LPS Concentration in Blood Samples

LPS concentrations in the blood were measured using PYROSTAR Neo (294-36731, Fujifilm Wako) and Control Standard Endotoxin (293-16541, Fujifilm Wako).

### 2.5. In Vitro Fertilization (IVF)

IVF was performed as previously described [24]. Young female ICR mice (3–6 weeks old) and adult male ICR mice (14–20 weeks old) were used for fertilization. Female mice received an i.p. injection of 7.5 IU eCG (serotropin, Asuka Animal Health Inc., Tokyo, Japan) to stimulate follicular growth, followed by an injection of 7.5 IU hCG (gonadotropin 3000, Asuka Animal Health Inc.) 49 h later to stimulate superovulation. At 15–16 h after hCG injection, the mice were euthanized by cervical dislocation and the cumulus–oocyte complexes (COCs) were collected from the mouse oviductal ampulla. Cumulus cells were removed using 0.3 mg/mL hyaluronidase in a droplet of 150 μL Toyoda-Yokoyama-Hosi (TYH) medium (DR01031, LSI Medience Inc., Tokyo, Japan) [25], which was covered with Paraffin Liquid (26114-75, Nacalai Tesque, Kyoto, Japan). The denuded oocytes were washed three times with droplets of 80 μL TYH medium. After washing, the denuded oocytes were inseminated in a droplet of 150 μL TYH medium with LPS concentrations of 0, 1, or 10 μg/mL as the fertilization medium. A pre-experiment was performed to finalize the LPS concentrations for IVF and final concentrations of 1 and 10 μg/mL in the fertilization medium were chosen. Sperms were recovered from the cauda epididymis and incubated to capacitation in a droplet of 150 μL TYH medium under a humidified atmosphere of 5% CO_2_ at 37 °C for 1 h. After incubation, the sperms were introduced into the fertilization medium with the denuded oocytes and incubated to achieve fertilization under a humidified atmosphere of 5% CO_2_ at 37 °C for 6 h. At 6 h after insemination, oocytes that exhibited two distinct pronuclei were considered to be fertilized. The fertilized eggs were washed four times with modified Whitten’s (mW) medium and transferred to droplets of 80 μL mW culture medium (DR01032-K, LSI Medience Inc.) [26,27] without LPS. The fertilization rate was calculated based on the number of collected oocytes and fertilized eggs. The embryonic development rate of the fertilized eggs was measured by observing the 2-cell, 4-cell, morula, and blastocyst stages. In total, 23 mice were used as oocytes donors for IVF. The IVF experiment was repeated six times.

### 2.6. LPS Administration In Vivo Using Mice

Female and male ICR mice (11–19 weeks old) were used to examine the effect of LPS exposure at fertilization on late embryonic development. Briefly, 25 female mice were mated with male mice overnight and the vaginal plug was examined the following morning (control group, n = 7; 10 µg/kg LPS group, n = 6; 100 µg/kg LPS group, n = 6; and 200 µg/kg LPS group, n = 6). The presence of a vaginal plug was classified as 0.5 dpc for the pregnant mouse. On the same day, mice received an i.p. of 0, 10, 100, or 200 μg/kg LPS. The pregnant mice were sacrificed at 18.5 dpc and the litter size, fetal resorption rate, placenta weight, fetal weight, and crown–rump length (CRL) were recorded. Furthermore, fetal tissue without internal organs was collected and used for gene expression analysis of cell cycle and apoptosis markers.

### 2.7. Gene Expression Analysis

Total RNA from tissue samples collected from the LPS administration experiments was extracted using TRIzol reagent (15596018, Thermo Fisher Scientific, Waltham, MA, USA). The total RNA concentration was measured using a Nanodrop (Thermo Fisher Scientific). Total RNA (1 µg) was treated with DNase and converted to cDNA with random primers (48190011, Thermo Fisher Scientific) and SuperScript Ⅱ (18064022, Thermo Fisher Scientific) using a GeneAtlas thermal cycler 482 (4990902, ASTEC, Fukuoka, Japan).

Real-time PCR was performed using the SsoAdvanced^TM^ Universal SYBR^®︎^ Green Supermix (1725271, Bio-Rad, Hercules, CA, USA) and LightCycler^®︎^ 96 system (05815916001, Roche, Basel, Switzerland) according to the manufacturer’s instructions. Each PCR reaction was performed at 95 °C for 30 s (denaturation), 95 °C for 10 s, and 35 cycles at 60 °C for 60 s (amplification). The primer sequences used are listed in Table 1. We used *β-actin* (ACTB) as the internal control, and the relative expression level of genes was calculated using the 2^−ΔΔCT^ method.

### 2.8. Formatting of Mathematical Components

The equation for color value is as follows:

ΔE*ab = (ΔL*^2^ + Δa*^2^ + Δb*^2^)/2 ΔL* = (each LPS group L*) − (control group L*) Δa* = (each LPS group a*) − (control group a*) Δb* = (each LPS group b*) − (control group b*)

### 2.9. Statistical Analysis

All statistical analyses were performed using the free software R version 4.2.2 (https://www.r-project.org/, access on 20 March 2023). Statistical analyses of LPS concentration, gene expression, and fetal development were conducted using one-way ANOVA with Dunnett’s test. A comparison of the mean fertilization and embryo developmental rate were performed using chi-squared tests. All data are expressed as the mean ± standard error of the mean (SEM). *p* < 0.05 was considered significant. Principal component analysis (PCA) was performed using the following factors: LPS concentration, fetal parameters (placental weight, fetal weight, and CRL), and gene expression (six genes).

## 3. Results

### 3.1. Maternal Effect of LPS Administration

**Figure 1:**
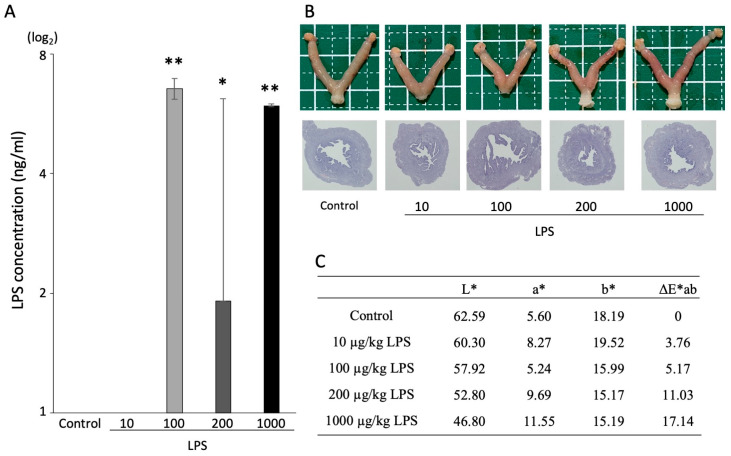
Effect of LPS on the immune response in the uterus. (**A**) Concentration of LPS in the plasma (n = 3). The values are shown as mean ±SEM. * *p* < 0.05, ** *p* < 0.01. (**B**) Histological examination of uteruses after administration of LPS. (**C**) Measurement of uterus color using spectrophotometry. L*: lightness, a*: redness, b*: yellowness.

**Figure 2:**
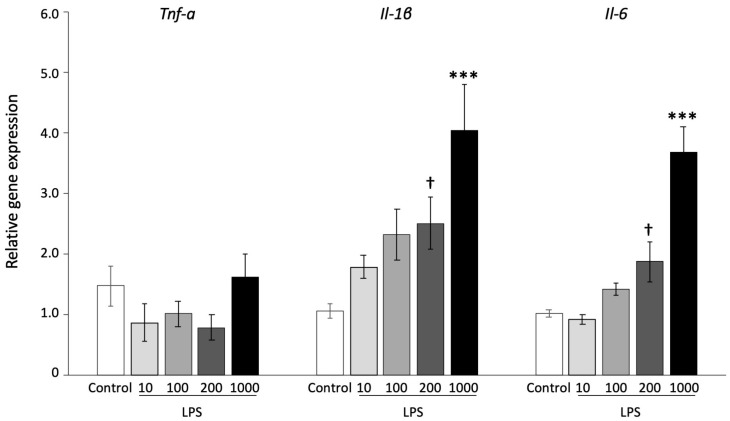
Gene expression of inflammatory cytokines in the uterus after administration of LPS. Mice were administered 0, 10, 100, 200, and 1000 µg/kg LPS and uteruses were collected after 6 h. The values are shown as mean ± SEM (n = 3). † *p* < 0.1, *** *p* < 0.001.

To examine the effect of LPS in uteruses, mice were administered 0, 10, 100, 200, or 1000 µg/kg LPS by i.p. injection. The high concentration of 1000 μg/kg LPS was administered to identify whether LPS clearly induced an acute inflammation. The administration of 100, 200, and 1000 µg/kg LPS significantly increased the concentration of LPS in plasma compared with the control group (Figure 1A, *p* < 0.01). To assess the inflammatory reaction, uterus color was measured by spectrophotometry. The value of L* (lightness) and a* (redness) displayed an inflammatory-like appearance in an LPS dose-dependent manner (Figure 1B,C). The expression of genes related to inflammatory responses was examined in the uteruses. Compared with the control group, the expression of *Tnf-α* did not significantly change following LPS administration, whereas that of *Il-1β* and *Il-6* demonstrated an increasing trend following the administration of 200 µg/kg LPS (*p* < 0.1) and significantly increased with 1000 µg/kg LPS (Figure 2, *p* < 0.001). 

### 3.2. Effect of LPS on Early Embryonic Development In Vitro

To investigate the effect of LPS exposure at fertilization on early development, we administered 0, 1, or 10 μg/mL LPS to denuded oocytes for fertilization. We calculated the development rate at the 2-cell, 4-cell, morula, and blastocyst stages (Table 2). The fertilization rate of the 1 and 10 μg/mL LPS groups were 78.8% and 76.9%, respectively, and significant differences were not observed compared with the control group. The development rate of the 1 μg/mL LPS group showed no differences compared with the control group at all development stages. However, the development rate of the 10 μg/mL LPS group displayed a decreasing trend in the 2-cell stage (95.0%, *p* < 0.1) compared with the control group (99.4%) and a significant decrease after the 4-cell stage (4-cell 83.0%, morula 71.0%, and blastocyst 62.0%, *p* < 0.05) compared with the control group (4-cell 93.6%, morula 84.9%, and blastocyst 82.0%).

### 3.3. Effect of LPS on Late Embryonic Development In Vivo

**Figure 3:**
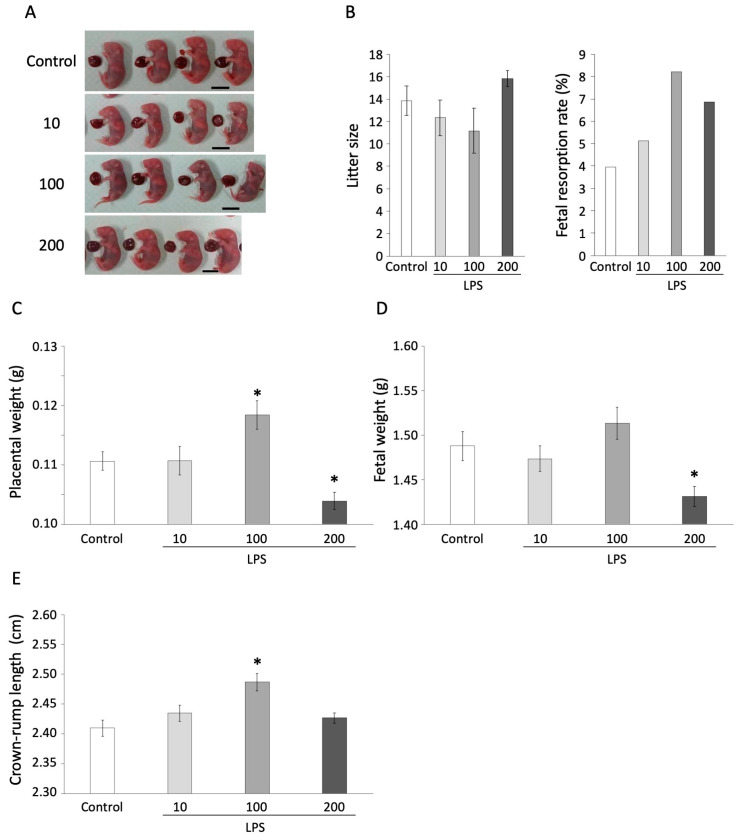
Effect of LPS for fetal development. Mice were administered 0, 10, 100, or 200 µg/kg LPS at 0.5 dpc (control n = 7, each LPS group n = 6). The fetal parameters were measured at 18.5 dpc. (**A**) Appearance of the litters in each group. (**B**) Litter size and fetal resorption rate. (**C**) Placental weight. (**D**) Fetal weight. (**E**) Crown–rump length. The values are shown as mean ± SEM (control n = 97, 10 µg/kg LPS n = 74, 100 µg/kg LPS n = 67, 200 µg/kg LPS n = 95) and * *p* < 0.05.

**Figure 4:**
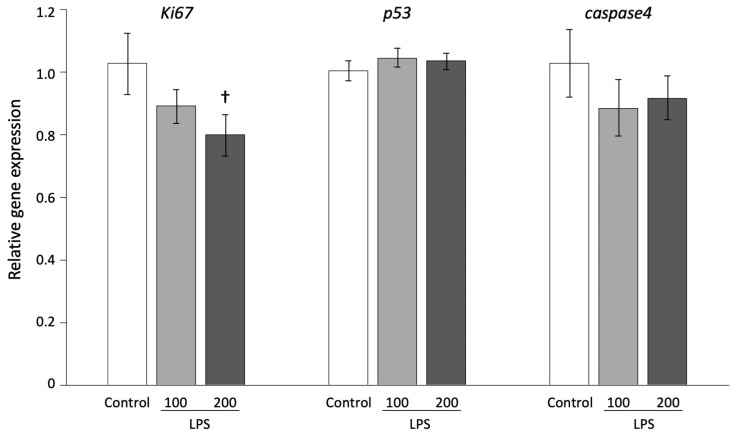
Gene expression of cell cycle and apoptosis markers in fetal tissue. The fetal tissues were collected at 18.5 dpc (n = 3) from litters of pregnant mice that had been administered 0, 100, and 200 µg/kg LPS. The values are shown as the mean ± SEM. † *p* < 0.1.

To examine the effect of LPS exposure at fertilization on embryonic development, we injected 0, 10, 100, or 200 µg/kg LPS into pregnant mice at 0.5 dpc. The pregnant mice were sacrificed at 18.5 dpc, and the effects of LPS exposure on litter size, fetal resorption rate, placental weight, fetal weight, and CRL before birth were examined (Figure 3A). The litter size and resorption rate were not affected at any concentration of LPS at 0.5 dpc (Figure 3B). However, 100 µg/kg LPS significantly increased the placental weight (0.118 ± 0.002 g) and CRL (2.487 ± 0.015 cm) compared with the control group (placental weight: 0.111 ± 0.002 g, fetal weight: 1.488 ± 0.016 g, CRL: 2.410 ± 0.014 cm) at 18.5 dpc (Figure 3C,E, *p* < 0.05). We also found that 200 µg/kg LPS significantly decreased the placental weight (0.104 ± 0.001 mg) and fetal weight (1.431 ± 0.011 g) compared with the control group (Figure 3C,D, *p* < 0.05). This indicated that the administration of LPS at 0.5 dpc affected late embryonic development. To reveal the molecular mechanisms underlying these effects, gene expression of the fetal tissue was analyzed after LPS administration. The expression of *Ki67*, which is related to the cell cycle, showed a decreasing tendency at 200 µg/kg LPS compared with the control group (Figure 4, *p* < 0.1), whereas the expression of *p53* and *caspase4*, which are related to apoptosis, were not affected by LPS.

### 3.4. Principal Component Analysis 

**Figure 5:**
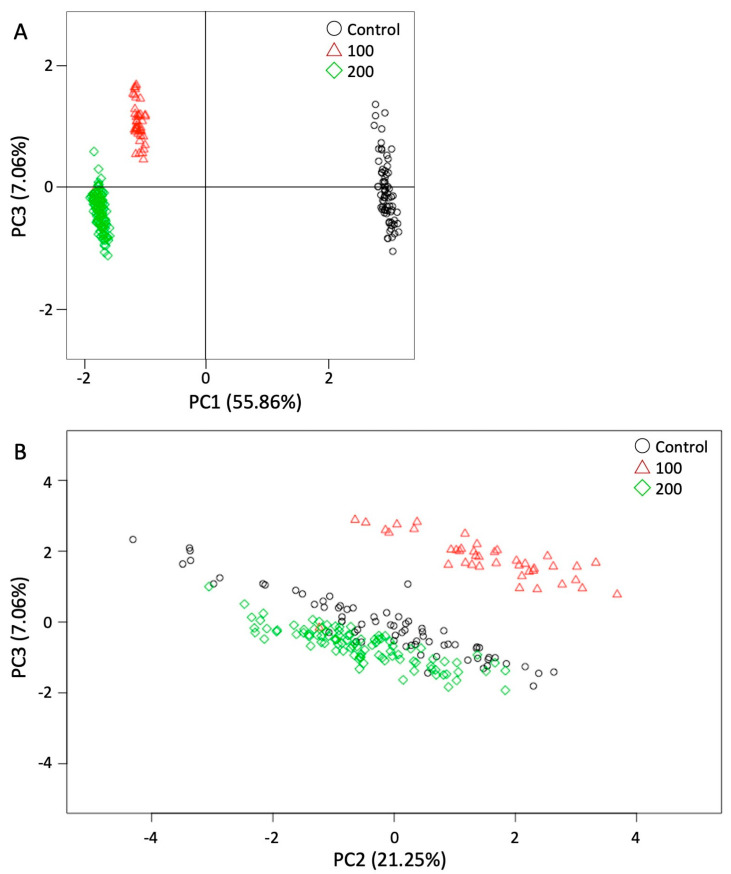
PCA performed using LPS concentration, fetal parameters, and gene expression. (**A**) Plot of PCA using PC1 and PC3. (**B**) Plot of PCA using PC2 and PC3. ○: control, △: 100 µg/kg LPS, ◇: 200 µg/kg LPS.

To comprehensively assess the effect of LPS administration at 0.5 dpc, we performed PCA using LPS concentration, fetal parameters, and gene expression in either the maternal uterus or fetal tissue. The three principal components (PCs) with eigenvalues >1.0 covered 90.79% of the cumulative proportion (PC1: 55.86%, PC2: 21.25%, and PC3: 7.06%). The PC1 factors mainly included the LPS concentration and *Il-1β*, *Il-6*, *Ki67*, *p53,* and *caspase4* expression (based on the largest loading values); PC2 included placental weight and CRL; and PC3 included fetal weight and *Tnf-α* expression (Table 3). Plotting PC1×PC3 showed a clear separation between 100 and 200 µg/kg LPS concentrations and the control group for fetal weight (Figure 5A). The plot of PC2 × PC3 showed separation between each LPS-administered group for placental weight, fetal weight, and CRL but not for LPS concentration (Figure 5B). 

## 4. Discussion

This study investigated the effects of LPS administration at fertilization on early embryonic and fetal development in pregnancy. LPS administration in mice increased gene expression of *Il-1β* and *Il-6* and induced inflammation in the uterus. Inflammatory uterus diseases, for example metritis and endometritis, are known to cause implantation failure and abnormal fetal development [28,29]. Therefore, embryonic development might be affected by inflammation of the reproductive tract during fertilization and implantation induced by LPS endotoxemia. Furthermore, previous IVF studies have reported that embryos from the 1-cell stage expressed TLR4, which is associated with pro-inflammatory cytokines [21,30]. Following LPS administration, these cells showed increased expression of the cytokine-related genes *Il-6* and *Tnf-α*, and these cytokines decreased embryonic development among mammals [21,31,32]. However, in this study we used denuded oocytes to analyze the direct effect of LPS on IVF. Our results showed that oocytes without a cumulus exhibited decreased embryonic development rates following LPS administration in vitro*,* which suggests that LPS directly affects embryonic development. 

Other studies have demonstrated that overexpression of the inflammatory cytokines *Il-1β* and *Il-6* induced cell proliferation in primary culture cells [33,34]. Furthermore, a previous study reported that LPS administration decreased the cell number of blastocysts, although the development rate of embryos did not change [35]. In this study, fetal development increased (particularly the placental weight and CRL) with 100 µg/kg LPS, whereas the placental and fetal weight decreased with 200 µg/kg LPS. Furthermore, 200 µg/kg LPS induced a decreasing trend in the gene expression of *Ki67* in the fetal tissue. In Figure 5A, the PCA results on the factors affected by LPS concentrations were clearly divided into three groups. In Figure 5B, the groups were affected by the fetal phenotype, such as the placental weight, fetal body weight, and CRL, without the LPS factor. As a result, LPS exposure at fertilization altered embryonic development and the immune response in uteruses, resulting in the phenomenon of abnormal fetal development. The difference in the effects with each LPS concentration was consistent with previous studies using animal models and cultured cells [36,37,38,39]. Previous reports have indicated that Ki67 is related to the initiation of the cell cycle [40,41]. The results presented here suggest that high concentrations of LPS affect the cell cycle and induce abnormal embryo development.

There are many studies on the impact of LPS on pregnancy, however, these were mainly performed in the late stages of pregnancy in mammals [22,37,39,42]. In contrast, in this study LPS was administered at fertilization (0.5 dpc), which is the early stage of embryogenesis. We did not observe changes in litter size and fetal resorption until 18.5 dpc, but placental and fetal weight and CRL were affected. This result suggested that LPS exposure at fertilization impacts fetal development until late-stage pregnancy. Therefore, we speculate that LPS might cause more critical damage related to fetal mortality at the late stages, when placentas have formed, than during the early stages of pregnancy. Inflammation of the placenta in humans has been reported to increase abnormal fetal phenotypes, for example diminished fetal growth, fetal death, and preterm birth [13]. However, we did not evaluate the expression of genes related to cytokines and the survival of placental tissue.

## 5. Conclusions

In summary, the present study demonstrated that LPS exposure at fertilization leads to the incidence of abnormal phenotypes during embryonic and fetal development in mice. The maternal endotoxin effect might impact the epigenetic inheritance of embryonic development from the early to late stages of pregnancy. This finding in the mouse model links the maternal environment before pregnancy to fetal development, and the effects are likely associated with infertility and developmental disorders. 

## Figures and Tables

**Table 1:** Primer pairs used in the gene expression analysis.

Gene	Primer		Size (bp)	Annealing Temperature (°C)	Accession No.
*Tnf-* *α*	F R	AAAGATGGGGGGCTTCCAGA GATGAGAGGGAGGCCATTTGG	157	60	NM_013693.3
*Il-1* *β*	F R	GCCACCTTTTGACAGTGATGAG AAGGTCCACGGGAAAGACAC	219	60	NM_008361.4
*Il-6*	F R	GGATACCACTCCCAACAGACC GGTACTCCAGAAGACCAGAGGAA	251	60	NM_001314054.1
*Ki67*	F R	GAGGCTGAGACATGGAGACATA TATCTGCAGAAAGGCCCTTGG	245	60	NM_001081117.2
*p53*	F R	TGGAGGAGTCACAGTCGGATAT ACACTCGGAGGGCTTCACTT	180	60	NM_011640.3
*caspase4*	F R	TAGACTCATTTCCTGCTTCCGG AGGTTGCCCGATCAATGGTG	128	60	NM_007609.3
*ACTB*	F R	CGTGCGTGACATCAAAGAGAA TGGATGCCACAGGATTCCAT	201	60	NM_007393.5

**Table 2:** Fertilization and development rate in IVF.

LPS Concentration (µg/mL)	No. of Denuded Oocyte	Development Stages				
		Oocytes Fertilized	2-Cell	4-Cell	Morula	Blastocyst
Control	213	172 80.8%	171 99.4%	161 93.6%	146 84.9%	141 82.0%
1	132	104 78.8%	104 100%	97 93.3%	83 79.8%	75 72.1%
10	130	100 76.9%	95 95.0% †	83 83.0% *	71 71.0% *	62 62.0% **

LPS was administered at 0, 1, and 10 µg/mL. † *p* < 0.1, * *p* < 0.05, ** *p* < 0.01.

**Table 3:** Proportion and variables of the three principal components (90.79%).

	PC1	PC2	PC3
Eigenvalue	5.586	2.125	1.368
Proportion (%)	55.86	21.25	7.06
Cumulative (%)	55.86	77.11	90.79
**Variables**			
LPS concentration	0.410	0.081	0.175
Placental weight	−0.037	−0.452	−0.047
Fetal weight	−0.084	−0.490	0.493
CRL	0.043	−0.567	0.303
*Tnf-* *α*	−0.049	−0.396	−0.669
*Il−1* *β*	0.422	−0.040	−0.330
*Il−6*	0.405	0.102	0.204
*Ki67*	−0.418	−0.050	−0.117
*p53*	0.386	−0.176	−0.265
*caspase4*	0.393	0.161	0.239

## Data Availability

The data presented in this study are available on request from the corresponding author.
